# Supplementary material for: Genome-Wide Identification of bHLH Transcription Factor in Medicago sativa in Response to Cold Stress
Source: Genes (Basel). 2022 Dec 15;13(12):2371. doi: 10.3390/genes13122371 (PMC9777957; doi:10.3390/genes13122371)
Supplement: Supplementary file 1 [file genes-13-02371-s001.zip › Comprehensive list of changes of genes-1998776 from Song Sheng.pdf]

Comprehensive list of changes

1. We carefully reviewed all of your corrections and responded in Word, such as "define abbreviations the first time, all gene and genus names into italic type, but do NOT change protein names into italic type", and correct all figure index in this paper. Other changes are as follows:
2. Include Jin Li and "School of Urban and Environment, Hunan University of Technology, Zhuzhou 412000, China" in the author list.
3. Delete “t20062073@csuft.edu.cn (G.L.); t20081077@csuft.edu.cn (L.J.)”, just leave the correspondence E-mail address;
4. At the end of “*2.3 Identification of Cold-Responsive MsbHLHs with RNA-Seq Data*”, add “Source code in R and RNA-seq data were provided in Supplemental data”;
5. At the end of “*2.5 Putative TFBSs Analysis in the Promoter Regions of MsbHLH Genes*”, add “Source code in R and TFBS data from PlantPAN3.0 were provided in Supplemental data”. This email will contain all of these data;
6. At Supplementary Materials, add “Source code, RNA-seq data, TFBS data were provided in Supplementary Data”;
7. At Author contributions, add Lin Li’s part who worked in “code review”, “resources” and “software”, L.L. provide numerous suggestions and arrange resources in this project and did review our code before submission.
8. Funding added, “This research was funded by “General items of Hunan Provincial Department of Education” grant number Project No.20C0623.”
