# Supplementary material for: Genome-Wide Identification of bHLH Transcription Factor in Medicago sativa in Response to Cold Stress
Source: Genes (Basel). 2022 Dec 15;13(12):2371. doi: 10.3390/genes13122371 (PMC9777957; doi:10.3390/genes13122371)
Supplement: Supplementary file 1 [file genes-13-02371-s001.zip › Supp Table 2.pdf]

Clade	TFBS.ID	Ratio	TF.family	Binding.sequence
1	TF_motif_seq_0024	2.889	(Motif sequence only)	tttttTGACG, CGTCAaccaa, tcaatTGACG, tttatTGACG, taaatTGACG
1	TF_motif_seq_0178	2.955	(Motif sequence only)	taaactatgattaGTAAG
1	TF_motif_seq_0181	2.955	(Motif sequence only)	ttctaaattttagTAAAC, gtgttcacgtatgTAAAC, taatccattacttTAAAC, cttttcatttgatTAAAC, gtttttattgatgTAAAC, gtgttcaagtatgTAAAC, GTTTAattcaatgaaata, ttttttttataggTAAAC
1	TF_motif_seq_0308	2.955	(Motif sequence only)	cGACGG, CCGTCg
1	TF_motif_seq_0467	2.955	(Motif sequence only)	AGATCgagg, AGATCgacg, AGATCgatg
1	TF_motif_seq_0473	2.955	(Motif sequence only)	caatCATTG, CAATGattg, CAATCattg, caatGATTG
1	TF_motif_seq_0178	3.611	(Motif sequence only)	taaactatgattaGTAAG
1	TF_motif_seq_0480	3.611	(Motif sequence only)	tcacTTGAG
1	TF_motif_seq_0462	4.213	(Motif sequence only)	ttagGGTTT, AAACCctaa
1	TF_motif_seq_0463	4.815	(Motif sequence only)	AAGACatag, AAGACgtat, ctagGTCTT

2	TF_motif_seq_0509	5.417	(Motif sequence only)	TTCCCtgtt
2	TF_motif_seq_0481	5.909	(Motif sequence only)	CTCCTaatt
2	TFmatrixID_0498	2.708	AP2	gTACGAag, caTCGTAc, tcTCGTAc, gTACGAtg
2	TFmatrixID_0622	5.417	AP2	TCGTAggtat, TCGTAggttg
2	TFmatrixID_0516	2.407	AP2; ERF	ataacatgggcttgTGTGCtg, atGCACAcattactaagctta
2	TFmatrixID_0082	2.955	AP2; ERF	agaCGGCGg, cCGCCGccg, tgaCGGCGt
2	TFmatrixID_0603	2.955	AP2; ERF	aGCCGTcatt, cGCCGTaatt, gaggACGGCt, attgACGGCg, aataACGGCt, tGCCGTaata
2	TFmatrixID_0039	3.611	AP2; ERF	accGCCGCcg
2	TFmatrixID_0054	3.611	AP2; ERF	cgCCGCCg
2	TFmatrixID_0056	3.611	AP2; ERF	cCGCCGccg
3	TFmatrixID_0061	3.611	AP2; ERF	CGCCGccg
3	TFmatrixID_0068	3.611	AP2; ERF	cgCCGGCg, cGCCGGcg
3	TFmatrixID_0069	3.611	AP2; ERF	acCGCCGccg
3	TFmatrixID_0075	3.611	AP2; ERF	cCGCCGcc

3	TFmatrixID_0081	3.611	AP2; ERF	acgCCGGCgt, acGCCGGcgt
3	TFmatrixID_0106	3.611	AP2; ERF	cgCCGGCg, cGCCGGcg
3	TFmatrixID_0600	3.611	AP2; ERF	ttgaCGGCGt
3	TFmatrixID_0147	3.095	AT-Hook	acaGGAAAag, ttTTTCCtgt, atTTTCCagt, acaGGAAAgc
3	TFmatrixID_0546	7.222	bZIP	tgACACGtcagc, catcaCGTGTac
3	TFmatrixID_0545	21.667	bZIP	ggCCACGacaaa
4	TFmatrixID_0212	3.611	C2H2	AGTTCaagt
4	TFmatrixID_0635	3.611	C2H2	ttGTGCActt, aggTGCACtc, tggTGCACtc, agGTGCActc, tgGTGCActc, ttgTGCACtt
4	TFmatrixID_0232	3.009	Dof	ACGTTata, aatAACGT, ACGTTatt, ACGTTatg
4	TF_motif_seq_0471	2.955	Homeodomain; HD-ZIP	caatGATTG, CAATCattg, CAATGattg, caatCATTG
4	TFmatrixID_0294	3.611	Homeodomain; TALE	ctTGACAt

4	TFmatrixID_0045	14.444	HSF	tttCTAGAac, ttTCTAGaaa, gttCTAGAac, gtTCTAGaac, ttTCTAGaac, tttCTAGAaa
4	TFmatrixID_0642	14.444	HSF	tttCTAGAaa, tttCTAGAac, gtTCTAGaac, ttTCTAGaac, ttTCTAGaaa, gttCTAGAac
4	TFmatrixID_0065	2.708	MADS box; MIKC	cctcCCAAAcatggccac, atgaCCAAAtaaggtgta, gatttcttcTTTGGtaat, agtcccatcTTTGGtaaa, gtttCCAAAtctagtaac, tttaCCAAAccttggaag, tttcctattTTTGGaaag, tcggacggtTTTGGtaaa, tttgcctgaTTTGGttgt, tgtcccatcTTTGGtaaa, ttttcttagTTTGGaaaa, aataactttTTTGGgaaa, attgCCAAAcctggtcca, ctttCCAAAtatggtttt
4	TFmatrixID_0104	2.708	MADS box; MIKC	ctttCCATAgatggtttt, ctttccaaaTATGGtttt

4	TFmatrixID_0470	2.708	MADS box; MIKC	tttgcctgaTTTGGttgt, gtttCCAAAtctagtaac, tttcctattTTTGGaaag, tcggacggtTTTGGtaaa, gatttcttcTTTGGtaat, aataactttTTTGGgaaa, ttttcttagTTTGGaaaa, ctttCCAAAtatggtttt, tttaCCAAAccttggaag, tgtcccatcTTTGGtaaa, atgaCCAAAtaaggtgta, agtcccatcTTTGGtaaa, attgCCAAAcctggtcca, cctcCCAAAcatggccac

5	TFmatrixID_0536	2.708	MADS box; MIKC	attgCCAAAcctggtcca, aataactttTTTGGgaaa, tgtcccatcTTTGGtaaa, ctttCCAAAtatggtttt, cctcCCAAAcatggccac, tttcctattTTTGGaaag, gatttcttcTTTGGtaat, ttttcttagTTTGGaaaa, tttaCCAAAccttggaag, tcggacggtTTTGGtaaa, agtcccatcTTTGGtaaa, atgaCCAAAtaaggtgta, gtttCCAAAtctagtaac, tttgcctgaTTTGGttgt
5	TFmatrixID_0529	3.611	MADS box; MIKC	cagtCCATAatcagaatt, ctttccaaaTATGGtttt
5	TFmatrixID_0355	3.009	Myb/SANT	tAACGGaaaa, cgatCCGTTa, tAACGGacct, tAACGGctgc, taaaCCGTTa, tAACGGatat

5	TFmatrixID_0519	3.611	Myb/SANT	ctacCTACCccag,<br>gtacCTACCtacc,<br>ttacCTACCaaca
5	TFmatrixID_0521	3.611	Myb/SANT;<br>MYB	ctacCTACCccag,<br>gtacCTACCtacc,<br>ttacCTACCaaca
5	TFmatrixID_0587	3.611	Myb/SANT;<br>MYB	ctacCTACCc,<br>gtacCTACCt,<br>ttacCTACCa
5	TFmatrixID_0347	10.833	Myb/SANT;<br>MYB	tAACCGct,<br>cAACCGtc,<br>tAACCGtt,<br>tAACCGcc,<br>aaCGGTTa
5	TFmatrixID_0017	3.095	Myb/SANT;<br>MYB; ARR-B	taGATACgca,<br>gaGATACgtt,<br>ctcGTATCtt,<br>aacGTATCtt,<br>agGATACgca
5	TF_motif_seq_0442	5.909	NAC	aacGCCGT
5	TFmatrixID_0385	7.222	NAC; NAM	tgACACGtcagc

6	TFmatrixID_0611	3.939	SBP	cacGTACGga,<br>aaCGTACgta,<br>aacGTACGta,<br>caCGTACgga,<br>atCGTACagt,<br>ccCGTACcca,<br>acaGTACGgt,<br>tggGTACGgt,<br>gcgGTACGat
6	TFmatrixID_0613	4.136	SBP	acGTACGgaa,<br>acGTACGtac,<br>tgaCGTACaa,<br>ccaCGTACgg,<br>aaaCGTACgt,<br>ggGTACGgtc,<br>cgGTACGatg,<br>agtCGTACtc
6	TFmatrixID_0415	5.417	Storekeeper	tttCCGGCct,<br>agGCCGGtct
6	TFmatrixID_0416	5.417	Storekeeper	gcGGCCGa,<br>gCGGCCga

6	TFmatrixID_0425	10.833	TCP	atGGGCCctt, ttgGGCCCaa, atgGGCCCtt, ttGGGCCcaa
6	TFmatrixID_0430	10.833	TCP	GTGGGacc
6	TFmatrixID_0433	10.833	TCP	GTGGGaccca, GTGGGacccc
6	TFmatrixID_0435	10.833	TCP	GTGGGacc
6	TFmatrixID_0439	10.833	TCP	GTGGGacc
6	TFmatrixID_0428	21.667	TCP	ttgGGCCCaa, ttGGGCCcaa
